# Data-Driven Model-Free Adaptive Control of Z-Source Inverters

**DOI:** 10.3390/s21227438

**Published:** 2021-11-09

**Authors:** Yasin Asadi, Amirhossein Ahmadi, Sasan Mohammadi, Ali Moradi Amani, Mousa Marzband, Behnam Mohammadi-ivatloo

**Affiliations:** 1 Department of Electrical Engineering, Shahid Bahonar University of Kerman, Kerman 7616913439, Iran; yasinasadi@eng.uk.ac.ir; 2 Department of Electrical and Software Engineering, University of Calgary, Calgary, AB T2N 1N4, Canada; amirhossein.ahmadi@ucalgary.ca; 3 Department of Electrical and Computer Engineering, Sharif University of Technology, Tehran 1136511155, Iran; mohammadi.sasan@ee.sharif.edu; 4 School of Engineering, Royal Melbourne Institute of Technology, Melbourne 2476, Australia; ali.moradiamani@rmit.edu.au; 5 Department of Mathematics, Physics and Electrical Engineering, Northumbria University, Newcastle NE7 7XA, UK; 6 Department of Electrical Engineering, University of Tabriz, Tabriz 5166616471, Iran

**Keywords:** Z-source, non-minimum phase, data-driven, model-free adaptive control, uncertainties

## Abstract

The universal paradigm shift towards green energy has accelerated the development of modern algorithms and technologies, among them converters such as Z-Source Inverters (ZSI) are playing an important role. ZSIs are single-stage inverters which are capable of performing both buck and boost operations through an impedance network that enables the shoot-through state. Despite all advantages, these inverters are associated with the non-minimum phase feature imposing heavy restrictions on their closed-loop response. Moreover, uncertainties such as parameter perturbation, unmodeled dynamics, and load disturbances may degrade their performance or even lead to instability, especially when model-based controllers are applied. To tackle these issues, a data-driven model-free adaptive controller is proposed in this paper which guarantees stability and the desired performance of the inverter in the presence of uncertainties. It performs the control action in two steps: First, a model of the system is updated using the current input and output signals of the system. Based on this updated model, the control action is re-tuned to achieve the desired performance. The convergence and stability of the proposed control system are proved in the Lyapunov sense. Experiments corroborate the effectiveness and superiority of the presented method over model-based controllers including PI, state feedback, and optimal robust linear quadratic integral controllers in terms of various metrics.

## 1. Introduction

Penetration of renewable energy resources has changed the structure of power systems toward distributed generation. To have a smooth and reliable transition in this paradigm shift, several technical and non-technical problems should be solved [1,2,3]. Inverters are one of the core components of future power grids with several applications in connecting batteries to grid, maximum power point tracking of solar panels, and smart grids [4]. Among different types of inverters, the buck-boost inverter is highly demanded due to lower stress on its components during operation as well as voltage increase/decrease capabilities [5,6]. However, traditional two-stage buck-boost inverters have the potential of short/open-circuit which avoidance requires time/overlap delays to be added in their operation. This indeed results in a higher cost, lower efficiency, more complexity, and a slight distortion in output waveforms [7].

To compensate for this drawback, Z-source inverters (ZSI) have been proposed which can operate in either voltage or current source modes [8]. They employ both buck and boost operations in one single-stage topology [9], using fewer switching devices [7], which results in a simple design and a high efficiency [10]. The shoot-through state of the ZSI is triggered by charging/discharging of capacitors and inductors in an X-shaped impedance circuit [11]. ZSIs have attracted a lot of research activities during recent years due to their applications in electric vehicles [12], wind power [13], and battery energy storage systems [14].

Several control methodologies, such as PI controllers [15], model predictive control (MPC) [16], fuzzy control [17], and sliding mode control [18], have been applied to improve their performance. While the experimental implementation of the fuzzy controller is also complicated [19], a LMI-based design method was introduced in Amirhossein et al. [20] to ensure Lyapunov stability of the inverter in the presence of uncertainties as well as keeping the desired level of disturbance rejection. The control and implementation of a bidirectional ZSI for a standalone hybrid system including PV, a diesel generator, and an energy storage system is explored in [21]. The current ripple of ZSI is analyzed and controlled in Shuai et al. [22] using an adjustable DC-link voltage and switching frequency strategy. A fractional-order PID controller is adopted in [23] for a bidirectional quasi-ZSI used in electric traction system, which is optimized using an ant colony optimization algorithm. Robust and fast control of a ZSI-based interior permanent magnet synchronous motor drive system is developed in Ghahderijani and Dehkordi [24] using the sliding mode control as the feedback controller empowered by a disturbance attenuation feed-forward compensator. Furthermore, a robust linear quadratic integral controller was recently introduced in Ahmadi et al. [11] for ZSIs subject to uncertainties, using the bat algorithm based on instantaneous exploitation [25].

Despite these advances, the Non-Minimum Phase (NMP) feature of ZSIs in addition to natural uncertainties which happen in different operating conditions severely impact the performance of linear control approaches. Although the NMP behavior can be modeled as a time delay, i.e., $1-\tau s=e^{-\tau s}$ [26,27], it causes both undershoot and overshoot in the transient response, escalates the harmonic distortions, and may lead to instability. The model-based predictive strategies, such as MPC, have shown the capability of dealing with NMP systems. However, they are not robust enough against variation of parameters.

The majority of control strategies that have been exerted on power electronic devices in the literature are based on a mathematical model of the system [11]. Generally, these models suffer from lack of unmodeled dynamics and precise knowledge of uncertainties, such as variation of load in electrical vehicles. It is well-known that components of renewable energy systems, such as ZSI, are subject to persistent uncertainties [28]. This means that model-based controllers that are designed based on these models may result in a poor performance in practice [29]. In other words, it is not possible to ascertain the practicality of most of the theoretical model-based results of a closed-loop control system (e.g., stability and convergence) [30]. One solution is using robust controllers like non-fragile $H_{\infty }$ multivariable PID controller [31], which requires complicated and conservative design procedure. However, thanks to advancements in information science and sensing technologies, a large amount of data has been generated and stored in many industrial processes, preserving all the useful state information of the process operations and equipment [32]. Therefore, the idea of compensating for incomplete mathematical models using process data has emerged. In fact, in cases where precise process models are not available, data can be used for controller design, prediction, diagnosis, and assessment of the industrial processes.

Inspired by this idea, the concept of data-driven control (DDC) has attracted much attention during recent years. It employs the Input and Output (I/O) measurements of the plant to prepare the appropriate control command [33]. Several data-driven methods have been developed so far including PID control, iterative feedback tuning, model-free adaptive control (MFAC), iterative learning, and virtual reference feedback control [34]. Although design and stability analysis of DDC methods is generally complicated, they have shown strong performance in tackling uncertainties, nonlinearities, and even cyber attacks to industrial systems [35]. It means that they can be appropriate solutions for control of ZSIs. Based on this, MFAC is applied to ZSI in this paper when uncertainties, including parameters perturbation, unmodelled dynamics, and external disturbances exist. Generally, MFAC is designed for effective control of discrete-time nonlinear systems [36,37], which is the case for ZSI. Several theoretical and experimental results for nonlinear multi-input-multi-output systems have been reported using MFAC, see, e.g., in Roman et al. [38], Xu et al. [39], Liang et al. [40]. This method is based on a dynamical linearization data model of the plant. A dynamic linearization method, incorporating the pseudo-partial derivative (PPD), places these data models at every dynamic operation point in the closed-loop system.

In this paper, ZSI is first simulated in different operating modes and its state-space model is obtained using input and output signals. As the model is updated online while the control system is running, this model includes uncertainties of the system as well. Then, a data-driven model-free adaptive controller is proposed for the system to guarantee stability and provide the desired performance. Indeed, this controller updates the control command based on the system model that is updated using latest input and out signals. The convergence and stability of the proposed control system are mathematically proved in the Lyapunov sense. To have a better evaluation, the performance of the proposed controller in managing nonlinearities, uncertainties, and the NMP behavior is compared with other controllers including PI, state-feedback (SF), and optimal robust linear quadratic integral (LQI) controller [11]. Simulation results show that the proposed controller successfully damps the external disturbance, tackles uncertainties, and provides a better transient response compared to its peers.

The remaining parts of this paper are structured as follows. Section 2 illustrates the circuit analysis and state-space model of the ZSI. In Section 3, a discussion of the proposed method is provided and the closed-loop stability of the control system is proved. Section 4 presents simulation results to establish the efficiency of the proposed controller. Finally, Section 5 briefly concludes the paper.

## 2. Circuit Analysis

**Figure 1:**
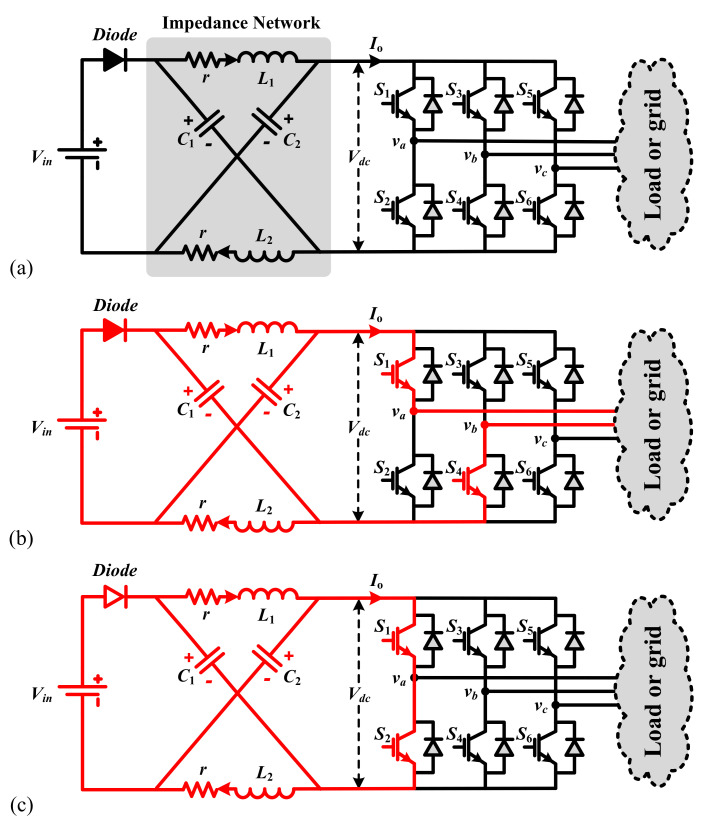
A voltage-source ZSI in (**a**) passive (**b**) non-shoot-through (**c**) shoot-through modes.

ZSI is a type of buck-boost power inverter with an extraordinary topology which does not require the well-known DC-DC converter bridge. Impedance Z-source networks efficiently facilitate the power conversion from the source to the load and are extensively applicable in numerous electric power conversion systems [41]. Figure 1 shows the topology of a three-phase voltage-fed ZSI constructed by the DC power source $V_{in}$; a Z-source network including $L_{1}$, $L_{2}$, $C_{1}$, $C_{2}$, and *r*; and a load which can be shown by $R_{o}$ and $L_{o}$. The desired value might be accomplished by changing the shoot-through duty ratio. Considering different switching states, the ZSI of Figure 1a can work in two modes: non-shoot-through and shoot-through modes. The state vector $x={\left[i_{L},v_{C},i_{o}\right]}^{T}$ can be used in deriving dynamical equations of these two modes, where $v_{c}$, $i_{L}$, and $i_{o}$ show the capacitor voltage, inductor current, and output current, respectively. The parasitic resistance of inductors is represented by *r*.

### 2.1. Shoot-Through Mode

**Figure 2:**
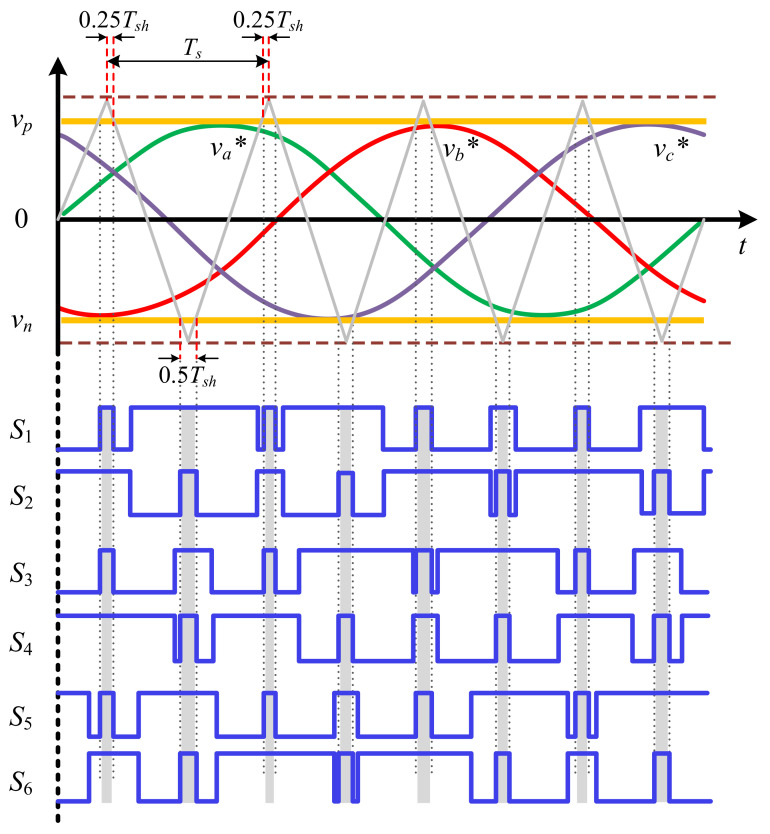
Three-phase simple boost PWM waveform.

In addition to the traditional six active and two zero states in the dynamical model, the ZSI incorporates a shoot-through zero state in order to increase voltage. The detailed operation of these states has been presented in Shen and Peng [42]. Generally, when the active states remain unchanged, only the zero states incorporate the shoot-through mode, and with a small modification in the zero states, an AC output voltage of the inverter will still resemble a traditional inverter besides the traditional PWM modulation methods. To have a better comprehension, in Figure 2 the overall method of boost PWM modulation is presented. The allocation of the shoot-through zero vectors for each phase eventually takes place with the total zero-vector time interval remaining unchanged. Therefore, interpolating shoot-through zero vectors enhances the DC-link voltage with no changes to the active-vector time. Here, the switching frequency is $f=1/T_{s}$ in which $T_{s}=T_{sh}+T_{n}$. The shoot-through and non-shoot-through intervals are shown by $T_{sh}$ and $T_{n}$, respectively, and the shoot-through duty cycle is $d=T_{sh}/\left(T_{sh}+T_{n}\right)$. As depicted in Figure 1c, in this mode, a mixture of lower and upper switches shortens the output terminals of the inverter. Furthermore, the diode is here reversely biased, meaning that the circuit is disconnected from the power system and inductors are charged by the stored energy in capacitors. Taking advantage of the shoot-through duty ratio and the energy transfer, the boosting capability of the ZSI has emerged. Here, the voltages throughout the inductors and DC-link are $v_{L}=v_{c}$ and $v_{dc}=0$. This mode is expressed in the following state-space model.

(1) $$\dot{x}=\underset{A_{sh}}{\underset{︸}{\left[\begin{array}{ccc} -\frac{r}{L} & \frac{1}{L} & 0\\ -\frac{1}{C} & 0 & 0\\ 0 & 0 & -\frac{R_{o}}{L_{o}} \end{array}\right]}}x$$

In the above equation, the effects of the input voltage power is omitted due to the diode inverse bias behavior.

### 2.2. Non-Shoot-Through Mode

In this mode, the energy is conveyed through the inductors and input source to reach the load and capacitors as shown in Figure 1b. The following mathematical model expresses the ZSI operation in this mode.

(2) $$\dot{x}=\underset{A_{n}}{\underset{︸}{\left[\begin{array}{ccc} -\frac{r}{L} & -\frac{1}{L} & 0\\ \frac{1}{C} & 0 & -\frac{1}{C}\\ 0 & \frac{2}{L_{o}} & -\frac{R_{o}}{L_{o}} \end{array}\right]}}x+\underset{B_{n}}{\underset{︸}{\left[\begin{array}{c} \frac{1}{L}\\ 0\\ -\frac{1}{L_{o}} \end{array}\right]}}V_{in}$$

Based on the circuit law, the voltages across inductors and the DC link are $V_{L}=V_{in}-V_{C}$ and $V_{dc}=V_{C}-V_{L}=2V_{C}-V_{in}$, respectively. The duty cycle in this mode is 1−d. By using the State-Space Averaging (SSA) technique, one may reach

(3) $$\dot{x}=\left(A_{n}\times \left(1-d\right)+A_{sh}\times d\right)x+\left(B_{n}\times \left(1-d\right)+B_{sh}\times d\right)V_{in}$$

Consequently, the global state-space model is gained by substituting (1) and (2) in (3) as follows:

(4) $$\dot{x}=\left[\begin{array}{ccc} -\frac{r}{L} & \frac{2d-1}{L} & 0\\ -\frac{2d-1}{C} & 0 & -\frac{1-d}{C}\\ 0 & \frac{2(1-d)}{C} & -\frac{R_{o}}{L_{o}} \end{array}\right]x+\left[\begin{array}{c} \frac{1-d}{L}\\ 0\\ -\frac{1-d}{L_{o}} \end{array}\right]V_{in}$$

Considering r=0, which is a known approximation in the literature [11], Equation (4) becomes

(5) $$V_{C}=\frac{1-D}{1-2D}V_{in}$$

in which *D* denotes the steady-state value of *d*. The average voltage across DC-link can be written as

(6) $$V_{dc}=\left(1-D\right)\times \left(2V_{C}-V_{in}\right)+D\times 0=\frac{1-D}{1-2D}V_{in}$$

Therefore, the relationship between the DC-link voltage and $V_{in}$ becomes

(7) $$v_{dc}=2V_{C}-V_{in}=\frac{1}{1-2D}V_{in}=BV_{in}$$

where B=1/(1−2D) is the boosting factor. As indicated in (7), the peak output voltage of the ZSI is illustrated as follows:

(8) $$v_{ac}=M\frac{v_{dc}}{2}=MB\frac{V_{in}}{2}$$

in which *M* is the modulation index. This indicates that, despite traditional inverters, ZSI could work in the buck mode and achieve the desired voltage by merely tuning an additional adjustable parameter like *B*.

## 3. The Proposed Controller

In order to deal with complexities of nonlinear systems, the use of the linearization techniques such as feedback linearization, Taylor’s linearization, and piecewise linearization [33] is popular. These approaches may result in sophisticated models which are not appropriate for controller design. For example, the orthogonal function-based approximation method would result in a model with many parameters or of a very high order. A model, which is linearized for data-driven control applications, should have a simple structure, moderate number of adjustable parameters, and convenient utilization of I/O data. Therefore, in this article, a new Compact Form Dynamic Linearization (CFDL) is employed in which the constraints play the role of inputs. In addition, the CFDL method is essentially concerned about how changes in the current input signal results in the variation of the output signal in the next time instant, thus appropriate for dynamical systems. A class of SISO nonlinear discrete-time systems is considered as

(9) $$y\left(h+1\right)=f(y\left(h\right),\ldots ,y\left(h-n_{y}\right),u\left(h\right),\ldots ,u\left(h-n_{u}\right))$$

where u(h)∈R and y(h)∈R are input and output of the system at time instant *h*, respectively; $n_{u}$ and $n_{y}$ are unknown positive integers; and $f\left(\cdot \right):R^{\left(n_{u}+n_{y}+2\right)}\to R$ is the unknown nonlinear function. Before expanding the CFDL model, some assumptions are required.

**Assumption** **1.** *The partial derivative of f(·) regarding the $\left(n_{y}+2\right)$th variable is continuous for all h with finite exceptions.*

**Assumption** **2.** *System (9) meets the generalized Lipschitz condition for all h with finite exceptions. This can be expressed as*

(10) $$|y\left(h_{1}+1\right)-y\left(h_{2}+1\right)|\leq b|u\left(h_{1}\right)-u\left(h_{2}\right)|$$

*where $u\left(h_{1}\right)\neq u\left(h_{2}\right)$ if $h_{1}\neq h_{2}$, $h_{1},h_{2}>=0$, and b is a positive constant. We also have $y\left(h_{i}+1\right)=f\left(y\left(h_{i}\right),\ldots ,y\left(h_{i}-n_{y}\right),u\left(h_{i}\right),\ldots ,u\left(h_{i}-n_{u}\right)\right)$ for i=1,2.*

These assumptions are adequately practical. **Assumption 1** is very reasonable in many control systems, and **Assumption 2** from an energy perspective means that the rate of change of energy in the system is limited by a factor of the input energy to the system. These assumptions are satisfied for many practical systems such as pressure or temperature control systems, power systems, and fluid level control systems. For the sake of abbreviation, the statement “for all *h* with finite exceptions” is omitted in the subsequent results.

**Theorem** **1.** *Suppose that system (9) satisfies the Assumptions 1 and 2, and |Δu(h)|≠0∀h. There exists a time-varying parameter $\vartheta_{c}\left(h\right)\in R$ which transforms the above system to the following CFDL:*

(11) $$\Delta y\left(h+1\right)=\vartheta_{c}\left(h\right)\Delta u\left(h\right)$$

*where $\vartheta_{c}\left(h\right)<\delta$ is bounded at any time h. The proof of this theorem was illustrated in detail in [37].*

**Remark** **1.** *Theorem 1 requires that |Δu(h)|≠0 for all h. In reality, if |Δu(h)|=0 occurs at a specific time, linearization can be applied after a time shift.*

All the nonlinear properties of the system are illustrated in $\vartheta_{c}\left(h\right)$. Obtaining the mathematical model of $\vartheta_{c}\left(h\right)$ directly can be very complex. However, its numerical behavior is obtained through nonlinear parameter recognition algorithms. For a SISO system such as y(h+1)=f(u(h)), the pseudo-partial derivative (PPD) represents the value of the derivative of the function *f* at a given point of *f* at a given point in the range of *h* to h+1. When PPD is upper bounded, no abrupt change is possible in the nonlinear function.

### 3.1. Algorithm of the Controller

The design algorithm of the MFAC is discussed in this subsection. A dynamic linear model corresponding to the nonlinear system is considered first. Then, the online input and output data is used to evaluate PDD, and the controller is designed such that a cost function is minimized. The cost function is

(12) $$J\left(u\left(h\right)\right)={\left|R\left(h+1\right)-y\left(h+1\right)\right|}^{2}+\lambda {\left|u\left(h\right)-u\left(h-1\right)\right|}^{2}$$

In this cost function, λ>0 represents the penalty factor that prevents the control signal from rapid changes, and *R* shows the reference signal. Substituting $y\left(h+1\right)=y\left(h\right)+\vartheta_{c}\left(h\right)\Delta u\left(h\right)$ in this cost function and using the Lagrange equation, the optimal control signal can be obtained, using derivation relative to *u*, as follows:

(13) $$u\left(h\right)=u\left(h-1\right)+\frac{\rho \vartheta_{c}\left(h\right)}{\lambda +|\vartheta_{c}{\left(h\right)|}^{2}}\left(R\left(h+1\right)-y\left(h\right)\right)$$

The parameter ρ∈(0,1] can make the algorithm more comprehensive, which is used to prove the stability in Hou and Jin [37].

**Remark** **2.** *As mentioned above, λ is a penalty factor in the cost function that prevents rapid changes in the control signal. As an essential tunable parameter in MFAC, the correct setting of λ reduces the tracking error.*

### 3.2. Estimation Algorithm of PPD

**Theorem1** shows that the the dynamic linearization model can be applied for nonlinear system (9) whenever Assumptions 1 and 2 are satisfied. In addition, Equation (16) shows that the optimal control signal can be accurately obtained if the PPD value is known. However, finding the exact PPD value from the model is difficult. An optimal PPD minimizes the cost function

(14) $$J\left(\vartheta_{c}\left(h\right)\right)=|y\left(h\right)-y\left(h-1\right)-\vartheta_{c}\left(h\right)\Delta u\left(h-1\right){|}^{2}+\mu |\vartheta_{c}\left(h\right)-{\hat{\vartheta }}_{c}\left(h-1\right){|}^{2}$$

in which μ>0 is a weight coefficient. From the optimal control theory, the value of ${\hat{\vartheta }}_{c}$ is obtained as

(15) $${\hat{\vartheta }}_{c}\left(h\right)={\hat{\vartheta }}_{c}\left(h-1\right)+\frac{\theta \Delta u(h-1)}{\mu +\Delta u{\left(h-1\right)}^{2}}\left(\Delta y\left(h\right)-{\hat{\vartheta }}_{c}\left(h-1\right)\Delta u\left(h-1\right)\right)$$

in which θ∈(0,1) denotes a step-size constant. Finally, the general form of CFDL-MFAC is given by

(16) $${\hat{\vartheta }}_{c}\left(h\right)={\hat{\vartheta }}_{c}\left(h-1\right)+\frac{\theta \Delta u(h-1)}{\mu +\Delta u{\left(h-1\right)}^{2}}\left(\Delta y\left(h\right)-{\hat{\vartheta }}_{c}\left(h-1\right)\Delta u\left(h-1\right)\right)$$

(17) $$u\left(h\right)=u\left(h-1\right)+\frac{\rho {\hat{\vartheta }}_{c}\left(h\right)}{\lambda +|{\hat{\vartheta }}_{c}{\left(h\right)|}^{2}}\left(R\left(h+1\right)-y\left(h\right)\right)$$

**Figure 3:**
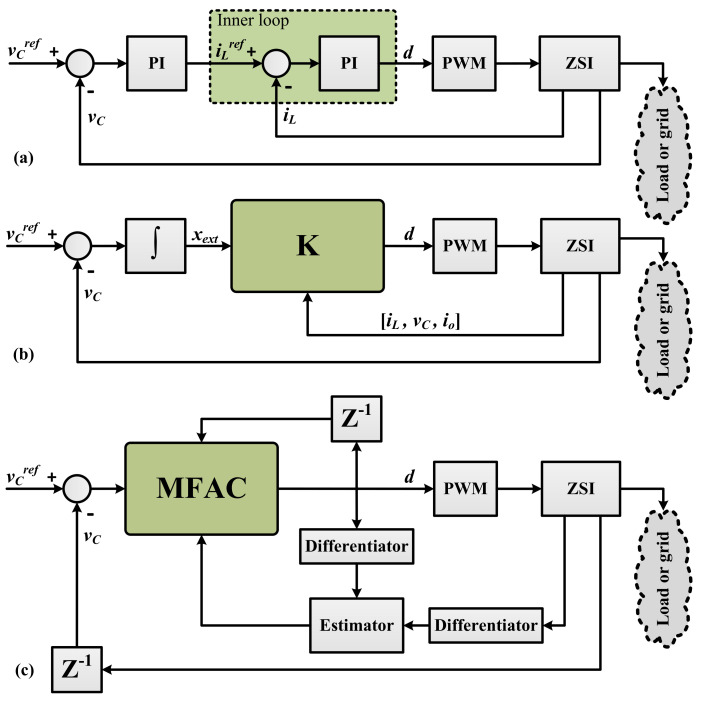
Double-loop control of a ZSI using (**a**) PI control, (**b**) state-feedback, and (**c**) proposed MFAC.

There is a reset mechanism to prevent the estimation algorithm from falling asleep and is implemented using ${\hat{\vartheta }}_{c}\left(h\right)={\hat{\vartheta }}_{c}\left(1\right)$, if ${\hat{\vartheta }}_{c}\left(h\right)\leq e$ or $sign\left({\hat{\vartheta }}_{c}\left(h\right)\right)\neq sign\left({\hat{\vartheta }}_{c}\left(1\right)\right)$. Figure 3c shows the block diagram of the proposed controller.

### 3.3. Stability Analysis

**Theorem** **2.** *Suppose the nonlinear system (9) is controlled using the controller (17) and the model update mechanism (16), under* ***Assumptions 1*** *and* ***2*** *. In regulation problem, that is, R(h+1)=R(h), there exists a constant $\lambda_{min}$ such that the regulation performance is satisfactory for all $\lambda >\lambda_{min}$. It means that $lim_{h\to \infty }\left|R\left(h+1\right)-y\left(h\right)\right|=0$.*

**Proof.**  We prove the theorem in two steps: In step 1, the boundedness of the pseudo-Jacobian matrix estimation ${\hat{\vartheta }}_{c}\left(h\right)$ is proved. Then, the convergence of the tracking error and the BIBO stability of the DDC system are shown.

Step 1: This step is already proved in Hou and Jin [37].

Step 2: In this step, we show that the output tracking error ε(h)=R(h)−y(h) is bounded. Considering the Lyapunov function $V\left(h\right)=\varepsilon^{T}\left(h\right)\varepsilon \left(h\right)$, one can achieve

(18) $$\Delta V\left(h+1\right)=\varepsilon^{T}\left(h+1\right)\varepsilon \left(h+1\right)-\varepsilon^{T}\left(h\right)\varepsilon \left(h\right)$$

where ε(h+1)=R(h+1)−y(h+1). For the regulation problem, i.e., when R(h+1)=R(h), (13) is expressed as

(19) $$\varepsilon \left(h+1\right)=R\left(h+1\right)-y\left(h\right)-\vartheta_{c}\left(h\right)\rho \frac{{\hat{\vartheta }}_{c}^{T}\left(h\right)\left(R\left(h+1\right)-y\left(h\right)\right)}{\lambda +|{\hat{\vartheta }}_{c}{\left(h\right)|}^{2}}$$

in which, ε(h+1)=H(h)ε(h) and $H\left(h\right)=I_{m}-\vartheta_{c}\left(h\right)\rho \frac{{\hat{\vartheta }}_{c}^{T}\left(h\right)}{\lambda +|{\hat{\vartheta }}_{c}{|}^{2}}$. Therefore,

(20) $$\begin{array}{cc} \Delta V(h+1)= & \varepsilon^{T}\left(h\right)H^{T}\left(h\right)H\left(h\right)\varepsilon \left(h\right)-\varepsilon^{T}\left(h\right)\varepsilon \left(h\right)\\ = & \varepsilon^{T}\left(h\right)\left[H^{T}\left(h\right)H\left(h\right)-I_{m}\right]\varepsilon \left(h\right) \end{array}$$

To prove the stability, it is sufficient to show that the |H(h)|<1. We have

(21) $$|I_{m}-\vartheta_{c}\left(h\right)\rho \frac{{\hat{\vartheta }}_{c}^{T}\left(h\right)}{\lambda +|{\hat{\vartheta }}_{c}{\left(h\right)|}^{2}}|<1$$

which leads to

(22) $$|\vartheta_{c}\left(h\right)\rho \frac{{\hat{\vartheta }}_{c}^{T}\left(h\right)}{\lambda +|{\hat{\vartheta }}_{c}{|}^{2}}\left|\leq |\rho |\right|\vartheta_{c}\left|\right|\frac{1}{2\sqrt{\lambda_{min}}}|$$

Considering the boundedness of ρ and δ, there exist a positive $\lambda_{min}$ such that

(23) $$|\vartheta_{c}\left(h\right)\rho \frac{{\hat{\vartheta }}_{c}^{T}\left(h\right)}{\lambda +|{\hat{\vartheta }}_{c}{|}^{2}}\left|\leq \right|\frac{\rho \delta }{2\sqrt{\lambda_{min}}}|<1$$

which completes the proof. □

## 4. Simulation Result

Suppose a load is supplied by an input voltage of 20 V through a ZSI that operates at 5 kHz and incorporates a simple-boost PWM. The load is a three-phase 50 Hz, 55 V (line), 5 A, and Y-connected with a 0.8 lagging power factor. System parameters are shown in Table 1 and are based on those in Hou and Jin [37]. Parametric uncertainties are considered in the load and the shoot-through duty cycle as $R_{o}\in \left[10,60\right]$ and D∈[0.3,1], respectively. The performance and robustness of the proposed controller are compared with a PI controller, a SF controller, and an optimal robust LQI controller. Figure 3a shows the PI controller within dual loop schemes. A simple integral action is used in the outer loop while the parameters of the inner PI controller are [11]

(24) $$PI=k_{i}/s,k_{i}=0.0564$$

The SF controller is designed as [11]

(25) K=[−0.00070.0031−0.071−0.0211]

(26) $$d\left(t\right)=-0.0007i_{L}+0.0031v_{C}-0.071i_{o}-0.021x_{ext}$$

The LQI controller can also be designed as [11]

(27) K=[0.62410.0153−0.1468−22.3607]

(28) $$d\left(t\right)=0.6241i_{L}+0.0153v_{C}-0.1468i_{o}-22.3607x_{ext}$$

The initial conditions of the proposed controller are u(1)=u(2)=u(3)=0, −y(1)=y(2)=y(3)=1, and $\vartheta_{c}\left(1\right)=20000$, and the step factors are ρ=0.6, θ=0.1, λ=0.5, and μ=0.2.

**Figure 4:**
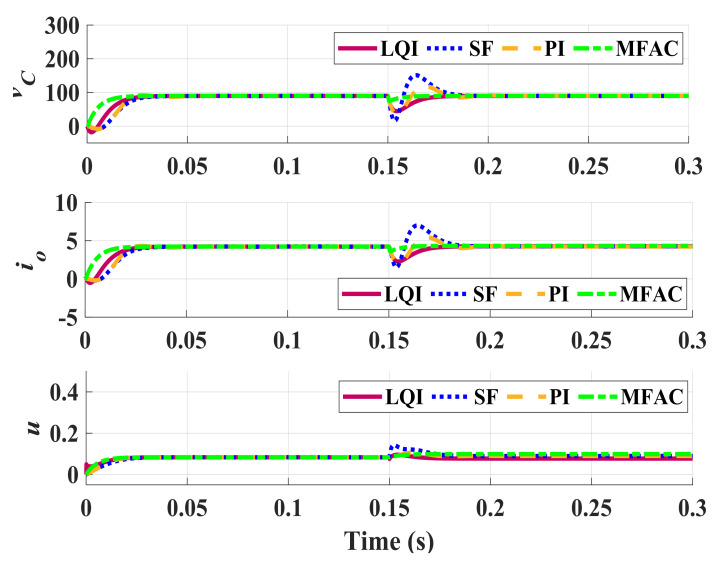
Nominal closed-loop response for a 4 A load disturbance (D=0.4347, $R_{o}=27$).

Performance of these controllers are compared with our proposed DDC one in the nominal condition, in the presence of perturbations, and also in the case of load fluctuations. Figure 4 shows the variations of the capacitor voltage $v_{C}$ and the output current $i_{o}$ caused by a 4A load disturbance. The nominal operating condition is considered to happen for D=0.4374 and $R_{o}=27$. This figure shows that all of the controllers respond in a stable regulatory manner with servomechanism and no constant error. However, in the undershoot behavior, the difference between SF, PI, and LQI controllers with the proposed method is notable since our DDC controller does not have any undershoot due to its prediction capability. Furthermore, in the disturbance rejection comparison, the proposed method is also powerful and damps the external disturbance in a short time. As is observed, the capacitor has reached the constant voltage of 89.8 V, slightly different from the expected 89.8146. The output current obtained is 4.236, again with only a slight difference from the expected 4.2362. Also, for the practical operation of the ZSI, the smoothness of the controllers is validated by TV metric. To better compare performance of these controllers, Table 2 summarizes the performance indices for these controllers.

**Figure 5:**
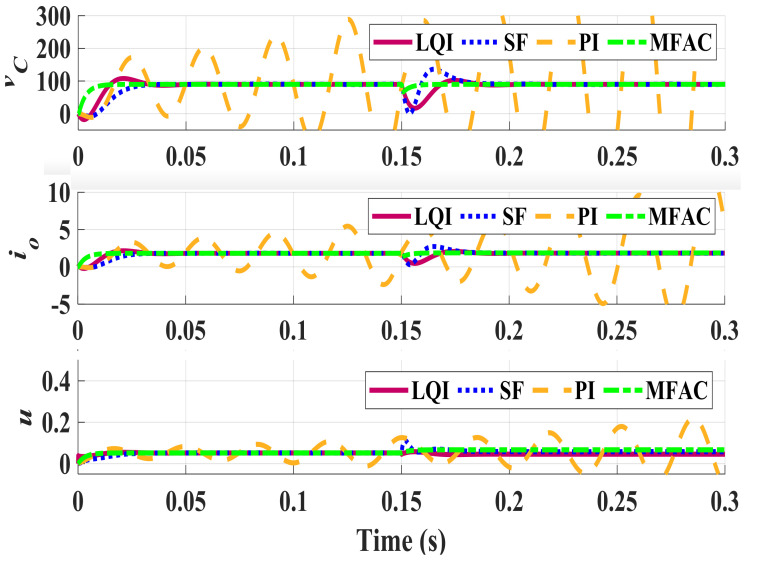
Perturbed closed-loop response for a 4 A load disturbance (D=0.45, $R_{o}=60$).

**Figure 6:**
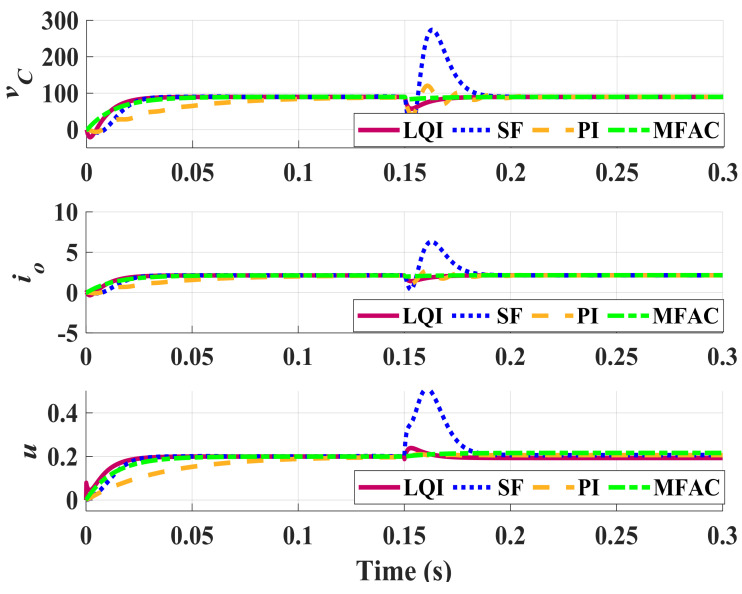
Perturbed closed-loop response for a 4 A load disturbance (D=0.4, $R_{o}=60$).

To compare the robustness of controllers against variations in plant parameters, the previous parameter setting is incorporated in stimulation with disturbances. In Figure 5, $v_{C}$ and $i_{o}$ are compared when the system parameters are perturbed to D=0.45 and $R_{o}=60$, comparing to the nominal condition D=0.4347 and $R_{o}=27$. A 4A load fluctuation is applied to the system and performance of different controllers are compared. Figure 5 shows stable operation of all controller, except PI, in this case. The proposed MFAC controller maintains the closed-loop response and provides a high robustness against parameter variations. Finally, the last simulation is performed in the perturbed condition (D=0.4, $R_{o}=60$) to investigate reliability. It could be seen in Figure 6 that SF and PI controllers no longer produce a satisfactory transient response and lead to low-frequency large or high-frequency small oscillations when rejecting disturbances. This means that a small parameter perturbation in *D* considerably impacts performance of these two controllers. The figure also shows that the proposed DDC is strong enough to reject this disturbance. This is mainly because of the model update mechanism and adaptive behavior of the controller. To better compare performance of these controllers, Table 3 and Table 4 summarize the performance indices for different perturbed conditions.

## 5. Conclusions

ZSIs have several potential applications in renewable-rich power grids. However, there are many technical challenges about them to be solved. They are non-minimum phase systems in nature, are subject to unexpected load variations and parameter uncertainties during their normal operation, and their semiconductor switches suffers from voltage stress if a control system causes a poor transient performance, such as overshoot. This means that linear and model-based control systems may fail to provide acceptable performance in all operating conditions. This paper proposed a data-driven model-free adaptive control for ZSIs. The controller had two parts: First, model of the system was updated based on the current input and output signals. Then, the controller tuned the control signal such that the error signal vanished. This meant that any uncertainties in the system would be reflected to the model, thus the controller could compensate for it. Stability of the proposed system was proved using the Lyapunov stability theory. Furthermore, the efficacy of the proposed controller was compared with PI, SF, and optimal robust LQI controllers. Simulation results showed that the proposed model-free approach performed more robust and reliable than its peers in the presence of uncertainties and load fluctuations. Despite this superior performance, the proposed method suffers from a wide range of parameters which makes the optimal tuning of the controller very sophisticated. Reducing the number of these parameters can be a matter of further research.

## Figures and Tables

**Table 1:** Parameters of the inverter in the nominal condition.

Parameter	Symbol	Value
Input voltage (V)	$V_{in}$	20
Shoot-through duty cycle	*D*	0.4374
Capacitor voltage (V)	$V_{C}$	89.8146
Inductor current (A)	$I_{L}$	19.05
Output current (A)	$I_{o}$	4.2362
Capacitor (μF)	*C*	92.25
Inductor (mH)	*L*	2.1
Coupling inductor (mH)	$L_{o}$	6.6
Load (Ω)	$R_{o}$	27
Parasitic resistance (Ω)	*r*	0.05
Switching frequency (KHz)	*f*	10

**Table 2:** Metrics of the nominal system (D=0.4374 and $R_{o}=27$).

		Servo Response		Regulatory Response	
**Controller**	**TV**	**IAE**	**MP(%)**	**IAE**	**Peak**
LQI	0.095	1.325	0	0.637	0
SF	0.083	1.562	0	26.456	62.027
PI	0.128	1.505	1.984	27.054	29.195
MFAC	0.016	0.869	0	0.112	0

**Table 3:** Metrics of the perturbed system (D=0.45 and $R_{o}=60$).

		Servo Response		Regulatory Response	
**Controller**	**TV**	**IAE**	**MP(%)**	**IAE**	**Peak**
LQI	0.066	1.656	0	1.197	0
SF	0.092	2.055	0	25.720	48.990
PI	0.968	10.256	72.331	8.651	106.560
MFAC	0.116	1.481	0	0.991	0

**Table 4:** Metrics of the perturbed system (D=0.4 and $R_{o}=60$).

		Servo Response		Regulatory Response	
**Controller**	**TV**	**IAE**	**MP(%)**	**IAE**	**Peak**
LQI	0.142	0.982	0	0.432	0
SF	0.120	1.409	0	2.999	195.893
PI	0.161	2.144	0	0.724	36.837
MFAC	0.116	0.123	0	0.157	0
